# Understanding the Effect of Impeller Configurations on Pullulan Production by *Aureobasidium Pullulans* RBF 4A3

**DOI:** 10.3389/fbioe.2019.00223

**Published:** 2019-09-26

**Authors:** Bhupender Kumar, Anjula Katoch, Gandham S. Prasad, Anirban Roy Choudhury

**Affiliations:** ^1^Biochemical Engineering Research and Process Development Centre (BERPDC), Institute of Microbial Technology (IMTECH), Council of Scientific and Industrial Research (CSIR), Chandigarh, India; ^2^University of Hyderabad, Hyderabad, India

**Keywords:** mass transfer, pullulan, impeller, *Aureobasidium pullulans*, exopolysaccharide

## Abstract

Mass transfer is one of the most important factors involved in viscous fermentation processes, like production of pullulan. Impellers play a crucial role in maintaining homogeneity and better mass transfer conditions during the fermentation process. The present study attempted to evaluate the efficiency of impellers with diverse configurations during pullulan fermentation. Initially, the mass transfer coefficients of 10 selected impellers were evaluated in an aqueous system. Among these, three impellers, namely, single helical ribbon, Rushton turbine, and Smith turbine impellers, were found to be more efficient and were further employed in the pullulan fermentation process. The results suggested that the single helical ribbon impeller was able to provide 24% higher pullulan production as compared to the Rushton turbine and Smith turbine impellers. The single helical ribbon was able to maintain the critical demand of dissolved oxygen in fermentation broth. Therefore, it may be commented that the single helical ribbon impeller configuration is suitable for higher production of pullulan during the fermentation process.

## Introduction

Pullulan is a neutral, water-soluble exopolysaccharide (EPS) produced by yeast-like fungus *Aureobasidium pullulans*. Structurally it consists of maltotriose subunits linked via α-1,4- and α-1,6-glycosidic bonds. Its unique structural composition proffers the biopolymer with several physiochemical properties, thus making it a suitable candidate for an array of applications in food, pharmaceuticals, agriculture, environment, cosmetics, and many more. After being recognized as a Generally Recognized as Safe (GRAS) product by the U.S. Food and Drug Administration (USFDA), it has been applied in the food industry as a gelling agent, emulsifier, stabilizer, and dietary fiber (Prajapati et al., 2013). Pullulan has the ability to form transparent, oil-resistant, and oxygen-impermeable films that can be used for food preservation (LeDuy et al., 2014; Unalan et al., 2015). These properties make pullulan acceptable for coating tablets and non-animal capsules (Moscovici, 2015). It is being widely used in pharmaceutical industries for targeted drug and gene delivery ascribable to its non-toxic and immunogenic properties. The excellent film-forming and rheological properties suggest the plausibility of pullulan being used in numerous high-value technological platforms (Ates, 2015).

Pullulan fermentation is an aerobic process and is often associated with the problem of high viscosity and poor mass transfer conditions. In viscous fermentations, better mass transfer and proper mixing of nutrients are maintained near the impeller swept zone, while due to high apparent viscosities at zones away from the impeller, the mass transfer conditions are poor. A good mass transfer can be achieved by understanding the oxygen consumption by cell and oxygen transfer rate in the system (Kawase et al., 1992; Garcia-Ochoa et al., 2010). Dixit et al. (2015) also reported enhanced productivity of pullulan by varying the impeller positioning favoring enhanced mass transfer in the fermenter (Dixit et al., 2015).

Majority of the literature focuses on increasing aeration and agitation rates individually for enhancing the mass transferability in the fermenter. Extensive research has been done to understand gas–liquid mass transfer in a stirred tank reactor (STR) with a water–air system. Generally, agitation and aeration are considered to be the two important variables for enhancing Volumetric mass transfer coefficient (K_L_a) values in a fermenter. In the case of aerobic fermentations, higher agitation rates support increased product mass attributable to better gas–liquid dispersion (Shu et al., 2019). However, increasing these variables beyond a critical point can be detrimental for microbial activity, as this can increase hydro-dynamic stress that may cause changes in cell morphology with an effect on cellular responses (Vlaev et al., 2013). A continuous increase of dissolved oxygen concentration and K_L_a in the fermenter with a gradual decline in cell biomass and pullulan concentration is observed on increasing aeration rate beyond 2 vvm (Roukas and Mantzouridou, 2001). Audet et al. (1996) reported that a high oxygen level during fermentation leads to more biomass rather than pullulan production and also affects the quality of EPS.

All these reports emphasize a need for the development of some different approach, apart from aeration and agitation, for resolving mass transfer problems in a bioreactor. Moreover, to the best of our knowledge, no studies have been conducted on understanding the correlation between mass transfer and pullulan production in employing different types of impellers. The present work was focused on understanding the effect of impeller configuration on mass transfer and pullulan fermentation in a fermenter along with varying aeration and agitation rates. Therefore, this clearly suggests that only enhancement of aeration and agitation rates will not resolve the problem of mass transfer in pullulan fermentation. Other than aeration and agitation, the configuration of impellers being used also plays a vital role in mass transfer distribution during viscous fermentations. The present study aims at predicting the effect of impeller configurations on mass transfer using the simulated Newtonian system. Considering their effect in the water–air system, they were further compared for their compatibility in viscous fermentation for pullulan production.

## Experimental Design

### Bioreactor Configuration

All the experiments were carried out in laboratory-scale 5 L STR (BioFlow 310, New Brunswick Scientific Co., Inc., Edison, NJ). The experiments were carried out in batch conditions at 28°C. The liquid phase was deionized water. The specifications of the 5 L STR are summarized in Table 1.

**Table 1:** Specification of stirred tank reactor.

**Descriptions**	**Value**
Body of vessel (material)	Glass
Internal diameter of vessel	25 cm
Vessel height	47 cm
Vessel aspect ratio	1.9:1
Working volume	3.3 L
Number of baffles	4
Baffle width	1 cm
Sparger type	Ring
Impeller diameter, for all impellers (in cm)	7.5
Ratio of impeller diameter to tank diameter	1:2

### Impellers

A total of 10 impellers (diameter, 7.5 cm) were employed in the present study, namely, the Rushton turbine (RT), double helical ribbon (DHR), hydrofoil (HDF), three blade segmented (TBS), Rushton turbine 45° (RT45), single helical ribbon (SHR), Rushton turbine curved blade (RTCB), pitch blade (PB), Smith turbine (SI), and screw helical ribbon (ScHR). All these impellers were tested initially in a 5 L fermenter with 3.5 L working volume of deionized water for evaluation of mass transfer rate from gas phase to aqueous phase in the fermenter. The impellers used for present study were designed with the same diameter. The values of aeration, agitation, and temperature were kept constant, viz, 0.5 vvm, 200 rpm, and 28 ± 1°C, respectively.

### Evaluation of Mass Transfer Coefficient (K_**L**_a) at Different Agitation and Aeration Rates

The oxygen mass transfer from gas to liquid phase in the fermenter system has utmost importance as it often serves as a controlling step in several biological processes. Henceforth, it is important to know K_L_a for carrying out the designing and scale-up of the bioprocess. In the present work, K_L_a from gas to liquid phase was measured by the dynamic gassing-out method (Bandyopadhyay et al., 1967). The mass balance for the dissolved oxygen in the liquid phase without occurrence of any biochemical process can be written as:

$$\text{dC}/\text{dT }={\text{ K}}_{\text{L}}\text{a}\left({\text{C}}^{*}-\text{C}\right)$$

where dC/dT, accumulation oxygen rate in liquid phase

C^*^, equilibrium dissolved oxygen concentration

C, oxygen concentration in liquid phase.

The dissolved oxygen concentration in the fermenter was measured using a Dissolved oxygen (DO) probe. Initially, the aqueous system was deoxygenated by feeding nitrogen gas, and then it was aerated again by passing air. The increase in DO was then measured for calculation of K_L_a.

### Application of Selected Impellers in Pullulan Fermentation Process

After studying the effect of 10 impellers in an aqueous system, impellers with a higher mass transfer coefficient were further selected to understand their effect on pullulan fermentation.

#### Microbial Strain and Inoculum Development

*Aureobasidium pullulans* RBF 4A3 was used for the present study. The organism was isolated from the flower *Caesulia axillaris*. The freshly grown cultures from YPD agar were transferred into a 250 ml flask containing inoculum medium composed of glucose (20 g/L), peptone (20 g/L), and yeast extract (10 g/L). The pH of the inoculum medium was set at 7 ± 0.2. The flask was incubated at 28°C for 15 h in a rotatory shaker at 200 rpm.

#### Pullulan Fermentation

The fermentation batches were carried out with different impellers, viz, RT, SI, and SHR. All fermentation experiments were carried out in a 5 L STR filled with 3.3 L fermentation medium composed of sucrose (15%), peptone (1%), and yeast extract (3%) (Choudhury et al., 2013; Sheng et al., 2016). The pH of the medium was calibrated up to 7 ± 0.2. Fermentation parameters like temperature, medium composition, pH, aeration rate, and agitation (2.0 vvm and 200 rpm) were kept constant for all the batches. The temperature was maintained at 28°C throughout the fermentation process, and pH was monitored continuously but was not controlled. The fermentation medium was inoculated with 5% freshly prepared seed culture.

#### Estimation of Residual Sugar

Residual sucrose was analyzed throughout the fermentation process from fermentation broth using biochemistry analyser YSI2001.

#### Biomass and EPS Estimation

Biomass and EPS estimation were performed as previously described by Choudhury et al. (2011). After every 12 h interval, 20 ml of fermentation broth was withdrawn and centrifuged at 10,000 rpm for 20 min, and then the obtained cell pellet was washed and resuspended in 0.85% NaCl solution and again centrifuged. The cell pellet was dried in an oven at 80°C until a constant weight was obtained. The supernatant obtained was then precipitated with ethanol at a 1:2 (v/v) ratio for pullulan estimation and kept overnight at 4°C. The precipitate thus obtained was centrifuged at 10,000 rpm for 20 min at 4°C and dried in an oven at 80°C until a constant weight was obtained.

### Statistical Analysis

All experiments were performed in triplicate. The average of data points was calculated along with standard deviation.

## Results and Discussion

### Screening of Impellers on the Basis of Mass Transfer in Aqueous System

**Figure 1:**
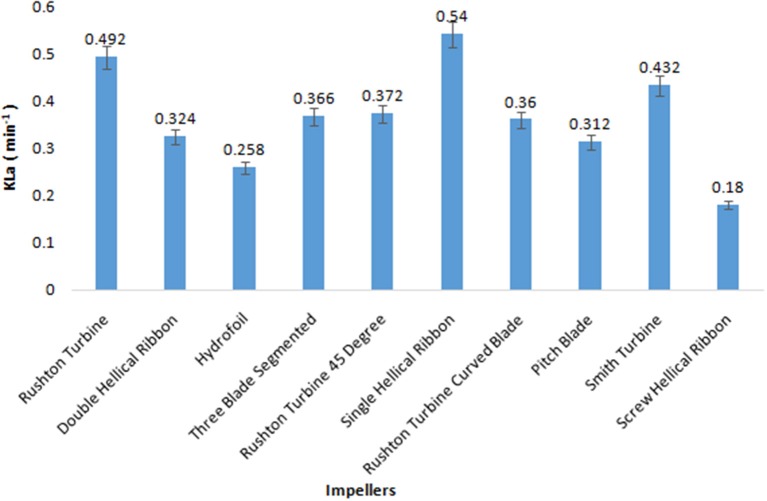
Volumetric mass transfer coefficient (K_L_a) values for various impellers.

The volumetric mass transfer efficiency of the 10 impellers was compared in a 5 L bioreactor with the aqueous system. During the course of screening of impellers, the aeration and agitation rates were kept constant at 0.5 vvm and 200 rpm, respectively, and values for K_L_a were recorded by the dynamic gassing-out method. Among the 10 impellers, SHR provided the highest K_L_a value of 0.54 min^−1^, followed by RT and SI with K_L_a values of 0.492 and 0.432 min^−1^, respectively (Figure 1). On the other hand, the K_L_a obtained for ScHR was found to be the lowest. In the case of aerobic fermentation, a higher volumetric mass transfer coefficient would ensure better productivity during fermentation. Hence, on the basis of obtained K_L_a values, three impellers, namely, SHR, RT, and SI, were selected for further studies.

**Figure 2:**
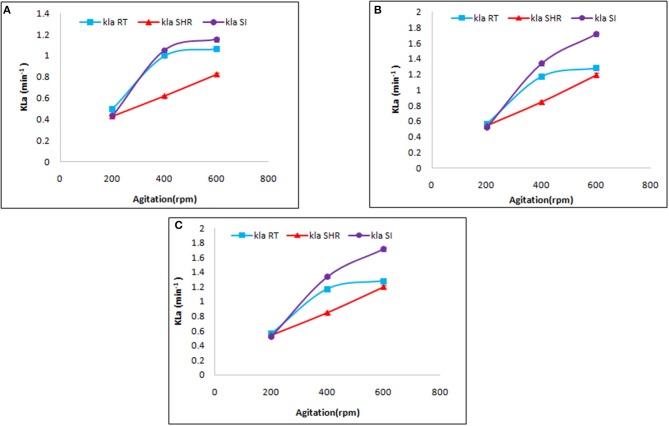
Effect of aeration and agitation rate on volumetric mass transfer in aqueous system at **(A)** 0.5 vvm, **(B)** 1 vvm, and **(C)** 1.5 vvm aeration. The graph represents the effect of single helical ribbon (SHR), Rushton turbine (RT), and Smith turbine (SI) impellers on mass transfer at varying rates of aeration (0.5, 1, and 1.5 vvm) and agitation (200, 400, and 600 rpm). SI was found to be the most efficient impeller amongst others.

Mass transfer by a specific impeller also depends on aeration and agitation. Therefore, the selected impellers were introduced to different values of aeration and agitation. At first, the selected impellers were studied to understand their influence on K_L_a at a different rate of aeration, viz, 0.5, 1.0, and 1.5 vvm, at varying agitation rates of 200, 400, and 600 rpm. As is evident from Figure 2, the selected impellers showed enhancement of K_L_a with a simultaneous increment of the aforementioned variables. The maximum K_L_a value was recorded for SI followed by RT and SHR, with values of 1.86, 1.488, and 1.356 min^−1^, respectively. These observations fall in line with the observations made in earlier reports (Devi and Kumar, 2017). In that case, the comparison was made between RT and curved blade (SI) impellers by using fluid dynamics. Earlier studies reported that the breaking of the air bubble is more in RT, which increases the area of the air bubble to enhance the oxygen transfer rate (Karimi et al., 2013). However, the maximum increment of 42% in K_L_a values was observed in the case of SHR on increasing agitation rate from 400 to 600 rpm at an aeration rate of 1.5 vvm, while the minimum increment of 12.2% was seen in the case of RT. Furthermore, on comparing the effect of aeration and agitation rates, it was observed that aeration solely could not increase K_L_a values, while an increase in the rate of agitation resulted in enhanced mass transfer. From Figure 2, it can be observed that for RT and SI impellers, an increase in agitation rate from 200 to 400 rpm showed a gradual increment in K_L_a values, while with a further increase in the rate of agitation from 400 to 600 rpm, both impellers slowly moved toward a plateau phase. The plot of K_L_a vs. aeration at constant agitation shows the same result. However, the case was different for SHR, where a continuous increase was observed, inferring that higher agitation and aeration values result in enhanced K_L_a values. The SHR represented an average enhancement on K_L_a with respect to the other two impellers. Correlation of aeration and agitation rates suggested that agitation plays a dominant role in K_L_a response as compared to aeration. This observation is supported by the studies of Kim et al. (2002) that reported an increase of K_L_a values from 0.003 to 0.029 s^−1^ with an increase in the rate of agitation from 200 to 700 rpm (Kim et al., 2002). Therefore, agitation plays an important role in the distribution of mass transfer, while aeration affects the retention time of oxygen flow with a change in gas velocity in the fermentation broth on varying aeration rates (Bandaiphet and Prasertsan, 2006).

### Effect of Impeller Geometries on Biomass and Pullulan Production

**Figure 3:**
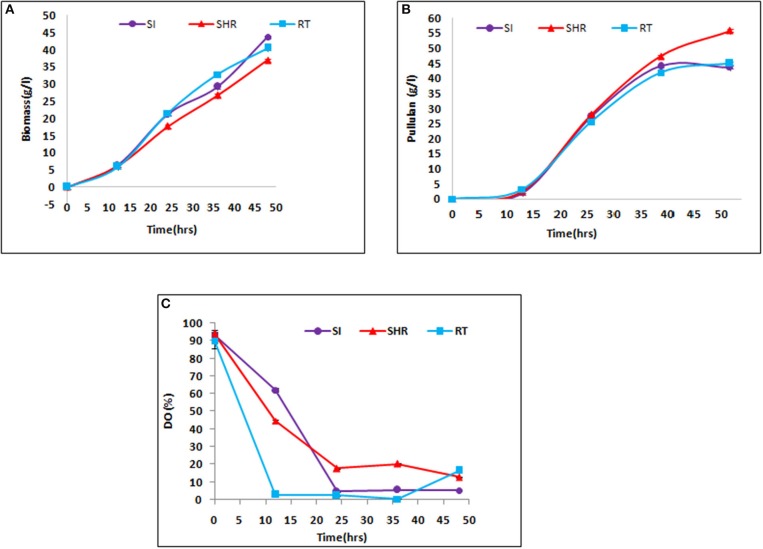
Kinetics of fermentative production of pullulan by *Aureobasidium pullulans* RBF 4A3 with different impeller **(A)** biomass production, **(B)** pullulan production, and **(C)** Dissolved Oxygen (DO) profile. The above graphs represent the comparative effect of three impellers, viz, SHR, RT, and SI, on biomass, pullulan production, and DO profile during 48 h of fermentation process. It is evident from **(A,B)** that SHR supports the least biomass formation as compared to the other two impellers, hence supporting the highest pullulan production of about 55 g/L.

The efficiency of all selected impellers, namely, SHR, SI, and RT, were tested in the case of viscous fermentation at certain operating conditions in pullulan fermentation. The maximum weight of biomass of 43.63 g/L was obtained at the end of 48 h, when the batch is operated at 1.5 vvm at 600 rpm with the SI impeller. It is prominent to note that the maximum mass transfer value was obtained in an air–water system, suggesting that higher K_L_a values supported the growth of *Aureobasidium pullulans*. From Figure 3A, it can be seen that SHR gave the lowest biomass concentration of 36.9 g/L. However, there is a gradual increase in biomass concentration in the case of SHR, while in the case of the other two impellers, biomass concentration starts moving toward a plateau after 36 h of fermentation, suggesting early onset of the stationary phase at higher K_L_a values. Cell growth is associated with substrate consumption. At the end of 48 h of fermentation time, complete sugar depletion was observed in all the batches with the tested impellers. However, the rate of sugar consumption varied along with impeller configurations, having different K_L_a values. In the case of higher K_L_a values, obtained from SI (1.86 min^−1^), the rate of sugar consumption by cells was initially fast, suggesting exponential growth of cells, and then after 30 h of fermentation time, sugar consumption rate gradually slows down, indicating the onset of the stationary phase in cells. Meanwhile, in the case of SHR, with the lowest K_L_a value of 1.4 min^−1^, the rate of sugar consumption by cells was initially slow and then gradually increased till the end of fermentation process, suggesting that cells remained in the exponential phase till 48 h of fermentation time. The result showed that enhanced value of the mass transfer coefficient increases the biomass concentration in the case of SI and RT. The biomass concentration had an increase of 16.32 and 7.72% in SI and RT, respectively, as compared with SHR. However, the duration of the exponential phase was higher in the case of SHR as compared to other impellers.

Pullulan production in all batches revealed a similar profile as for biomass concentration. Pullulan concentration increased throughout the fermentation process for all three impellers. However, the maximum pullulan concentration of 55.82 g/L was obtained at the end of the fermentation process in the case of SHR, wherein the lowest biomass concentration was reported (Figure 3B). From these results, it can be inferred that higher initial K_L_a facilitates cell growth, while lower K_L_a values support EPS production in the case of pullulan fermentation. Among the selected impellers, the RT and SI impellers showed an exponential increase in pullulan concentration up to 36 h of the fermentation period, after which no significant increase in pullulan concentration was observed. Interestingly, it was observed in the case of SHR that there was a gradual increase in pullulan production till the end of the batch.

Higher pullulan yield and lower biomass production were observed with the SHR, which may be attributable to a maintained DO level at optimum concentration in the fermentation broth as compared to RI and SI impellers, thereby producing biomass at the cost of EPS production Figure 3C. In the case of SHR, DO level dropped from 100 to 17.7% within 24 h and, after that, remained at up to 12.8% through the completion of the batch. In the case of RT, DO level dropped from 100 to 2.4% within 24 h and, after that, remained at up to 0.3% within 36 h and then increased to 16.5% at the completion of the batch. In the case of the SI, DO dropped from 100 to 4.6% within 24 h and, after that, remained at up to 5.3% through the completion of the batch. The high yield of pullulan obtained with the SHR may also be correlated with the reduction in shear rate and good circulation in the fermentation broth. These observations suggest that SHR provides better mixing and thus provides better homogeneity in the system and maintains the essential oxygen concentration throughout the fermentation process, therefore supporting higher pullulan production than RT and SI impellers. Gibson and Coughlin (2002) reported that the higher DO level with the RT produced high biomass but less concentration of EPS, as compared to the marine propeller (Gibson and Coughlin, 2002).

The results exhibited that there was a significant difference in the DO concentration profile. In the case of RT and SI, the rate of consumption of oxygen was higher as compared with SHR. RT and SI exhibited higher Y_x/s_ values, 0.269 and 0.291, respectively, as compared with SHR, which exhibited a lower Y_x/s_ value of 0.246. The productivity of SHR (1.162 g/L.h) was higher as compared with RT and SI (0.936 and 0.908, respectively). With higher Y_x/s_ values in the case of RT and SI, a sudden increase in biomass was observed with the reduced productivity (Table 2). These observations revealed that SHR with a gradual increase in K_L_a imparted the optimum level of DO concentration to prolong the exponential conditions for biomass growth. Hence, higher pullulan production was reported in the case of SHR as compared with RT and SI.

**Table 2:** Fermentation kinetics of pullulan by *Aureobasidium pullulans* RBF 4A3 with different impeller.

**Impeller type**	**K_**L**_a**	**Final BM (g/L)**	**Final EPS (g/l)**	**Yx/s**	**Yp/s**	**Yp/x**	**Productivity (g/l/h)**
RT	1.5	40.46	44.95	0.269	0.299	1.11	0.936
SI	1.9	43.69	43.63	0.291	0.29	0.998	0.908
SHR	1.4	37.56	55.82	0.246	0.372	1.512	1.162

*EPS, exopolysaccharide; RT, Rushton turbine; SI, Smith turbine; SHR, single helical ribbon; BM, Biomass*.

## Conclusion

The present study showed some important aspects of the effect of impeller types on mass transfer and pullulan production by *Aureobasidium pullulans* in a stirred tank bioreactor. The mass transfer value increases with an increase in aeration and agitation rates in the case of all types of impellers used in the present work. The comparative data analysis suggested that higher K_L_a values support cell growth of *Aureobasidium pullulans*, while optimum K_L_a values support pullulan production, which further suggested that it's not only K_L_a that affects pullulan production, but maintenance of critical oxygen concentration in fermentation broth is also an important factor for obtaining higher productivities of pullulan. Basically, pullulan production mainly depends on the rate of dissolved oxygen consumption by cells; the slower the rate of oxygen uptake by cells, the lower the biomass concentration, and eventually, the higher will be the pullulan production. The obtained productivity for pullulan suggested SHR to be the best impeller type for sustainable enhancement of bioreactor performance in the case of pullulan production, which also maintains the optimum level of DO.

## Data Availability Statement

The datasets generated for this study are available on request to the corresponding author.

## Author Contributions

BK and AK performed the experiments and drafted the manuscript. AR, BK, and AK contributed in the design of the experiments and analysis of the data. GP provided inputs in the same. AR conceived, designed, and supervised the complete study.

### Conflict of Interest

The authors declare that the research was conducted in the absence of any commercial or financial relationships that could be construed as a potential conflict of interest.
